# The influence of crisis on policy formulation: the case of alcohol regulation in South Africa during COVID-19 (2020–21)

**DOI:** 10.1093/heapol/czae055

**Published:** 2024-06-28

**Authors:** Mumta Hargovan, Leslie London, Marsha Orgill

**Affiliations:** School of Public Health, University of Cape Town, Observatory, Cape Town 7925, South Africa; School of Public Health, University of Cape Town, Observatory, Cape Town 7925, South Africa; The Children’s Institute, University of Cape Town, Rondebosch, Cape Town 7700, South Africa

**Keywords:** Policy formulation process, alcohol policy, crisis context, South Africa

## Abstract

This study contributes to a neglected aspect of health policy analysis: policy formulation processes. Context is central to the policy cycle, yet the influence of crises on policy formulation is underrepresented in the health policy literature in low- and middle-income countries (LMICs). This paper analyses a detailed case study of how the COVID-19 crisis influenced policy formulation processes for the regulation of alcohol in South Africa, as part of COVID-19 control measures, in 2020 and 2021. It provides a picture of the policy context, specifically considering the extent to which the crisis influenced the position and power of actors, and policy content. Qualitative data were collected from nine key informant interviews and 127 documents. Data were analysed using thematic content analysis. A policy formulation conceptual framework was applied as a lens to describe complex policy formulation processes. The study revealed that the perceived urgency of the pandemic prompted a heightened sense of awareness of alcohol-related trauma as a known, preventable threat to public health system capacity. This enabled a high degree of innovation among decision-makers in the generation of alternative alcohol policy content. Within the context of uncertainty, epistemic and experiential policy learning drove rapid, adaptive cycles of policy formulation, demonstrating the importance of historical and emerging public health evidence in crisis-driven decision-making. Within the context of centralization and limited opportunities for stakeholder participation, non-state actors mobilized to influence policy through the public arena. The paper concludes that crisis-driven policy formulation processes are shaped by abrupt redistributions of power among policy actors and the dynamic interplay of evolving economic, political and public health priorities. Understanding the complexity of the local policy context may allow actors to navigate opportunities for public health-oriented alcohol policy reforms in South Africa and other LMICs.

Key Messages

- Preventable public health burdens, including alcohol harms, may gain renewed policy attention and prioritization in health crisis contexts.
- Although tensions between public health evidence and values, political priorities and normative economic orientations persist during crisis-driven policy formulation processes, there may be a redistribution of power between actors across new and existing decision-making platforms.
- Actors may cast doubt on scientific evidence to support competing commercial agendas in crisis contexts, pointing to the need to address and manage conflicts of interest in academic and media platforms.
- Within a crisis context of urgency, uncertainty and centralization, policy cycles are compressed and non-linear, moving rapidly back and forth between policy formulation processes and implementation.

## Introduction

The COVID-19 pandemic has drawn attention to the links between alcohol control, population health and the public health system in South Africa (SA). SA ranks among countries with the most hazardous patterns of drinking globally (World Health Organization, 2018). While only a third of the population consumes alcohol, 43% of alcohol consumers report binge-drinking, and this is associated with a range of harms and costs (Matzopoulos *et al*., 2014; Vellios *et al*., 2018).

Alcohol is the third-largest contributor to disability and death in SA, with intentional injuries (interpersonal violence and suicide) forming the leading cause of alcohol-related harms and costs (Azar *et al*., 2010; Corrigall and Matzopoulos, 2012). Alcohol is further implicated as an intermediary in causal pathways across SA’s four concurrent epidemics: injuries and trauma, including interpersonal and gender-based violence and drunk driving accidents, infectious diseases, including HIV and Tuberculosis (TB), non-communicable diseases (NCD), including mental health disorders, and maternal and child conditions (Coovadia *et al*., 2009; Schmidt *et al*., 2010; Craig *et al*., 2023). The estimated prevalence of alcohol use disorders, including alcohol dependence and harmful use of alcohol, is 7%, nearly double the WHO African regional average (World Health Organization, 2018).

Economically vulnerable and marginalized communities in SA bear the brunt of alcohol-related harms and costs (Stein and Myers, 2016; Fontes Marx *et al*., 2019). This includes communities that have historically been affected by the ‘dop system’; an exploitative colonial and apartheid labour practice involving the compensation of farmworkers with rations of crude wine (London, 1999; Mager, 2004). However, direct and indirect adverse effects extend to all social and economic groups (Matzopoulos *et al*., 2014). Harmful alcohol use, and in particular, alcohol-related injuries and trauma, contribute significantly to bed occupancy in public health facilities and constitute a significant drain on limited healthcare staff and resources (Matzopoulos *et al*., 2014; Hardcastle *et al*., 2016; Schuurman *et al*., 2015).

The World Health Organization’s (WHO) strategy to reduce the harmful use of alcohol outlines evidence-based policy interventions in three domains: restrictions on access to retail alcohol, increasing taxation and bans on alcohol advertising (World Health Organisation, 2010). However, policy processes to update SA’s national Liquor Act of 2003 (Republic Of South Africa, 2004) have been characterized by inertia and indecision. Policy processes were marred by a lack of cohesion among state sectors and the influence of competing commercial interests (Parry, 2010; Parry *et al*., 2014; Bertscher *et al*., 2018).

In 2013, a bill proposing to ban alcohol advertising and sports sponsorships was drafted, but never gazetted or released for public comment (Bertscher *et al*., 2018). In 2016, an amendment to the National Liquor Act was approved by Cabinet and released for public comment, but the policy process stalled, with parliamentary review still pending (Zulu, 2021). The objectives of the amendment bill included: limiting alcohol advertising, restricting trading days and hours for alcohol manufacture and distribution, raising the legal drinking age to 21 years, increasing regulations on licensing and reducing outlet density (Department of Trade and Industry, 2016).

The onset of the COVID-19 pandemic in 2020 transformed the national policy context for alcohol regulation (Matzopoulos *et al*., 2020; Rehm *et al*., 2020). The Disaster Management Act of 2002 (DMA) provided the legislative basis for a series of temporary alcohol sales bans, intended to reduce rates of alcohol-related trauma, thus freeing up hospital beds, healthcare resources and healthcare worker capacity to respond to the crisis (Health24, 2020). Following an initial ban on alcohol sales in March 2020, lasting over 2 months, regulations were adjusted frequently over the course of the pandemic in 2020 and 2021^1^ (Table 1).

**Table 1.** Timeline of key events in alcohol regulation during the COVID-19 pandemic (2020–21)

Date	Events	Alcohol regulations
30 Jan 2020	WHO declares a Public Health Emergency of International Concern (PHEIC)	
5 Mar 2020	SA reported its first case of COVID-19	
15 Mar 2020	National State of Disaster declared in SA (came into effect on 17 Mar 2020 and ended on 5 April 2022) NCCC established	
19 Mar 2020	Initial COVID-19 regulations^19^	Partial restrictions on alcohol sales^20^
26 Mar 2020	Lockdown begins Ministerial Advisory Committee on COVID-19 appointed	First complete ban on alcohol sales (2 months, 5 days)
1 Jun 2020		Partial restrictions on alcohol sales
13 Jul 2020	First wave of the pandemic^21^	Second complete ban on alcohol sales (1 month, 5 days)
18 Aug 2020		Partial restrictions on alcohol sales
16 Sep 2020		No restrictions on alcohol sales^22^
3 Dec 2020		Partial restrictions on alcohol sales
28 Dec 2020	Second wave of the pandemic Festive period, which is known for high trauma rates in SA	Third complete ban on alcohol sales (1 month, 4 days)
1 Feb 2021		Partial restrictions on alcohol sales
17 Feb 2021	National COVID-19 vaccination program commenced	Partial restrictions on alcohol sales
1 Mar 2021		No restrictions on alcohol sales
2 Apr 2021	Amended lockdown regulations over the Easter period begins	Partial restrictions on alcohol sales
6 Apr 2021		No restrictions on alcohol sales
28 Jun 2021	Third wave of the pandemic	Fourth complete ban on alcohol sales (28 days)
12 Jul 2021	Civil unrest in Gauteng and Kwa-Zulu Natal provinces	Fourth complete ban on alcohol sales
26 Jul 2021		Partial restrictions on alcohol sales
1 Oct 2021		No restrictions on alcohol sales
1 Nov 2021	Municipal elections	No restrictions on alcohol sales
28 Nov 2021	Fourth wave of the pandemic No change to national regulations	No restrictions on alcohol sales
30 Dec 2021	Reduced national regulations and Festive period, which is known for high trauma rates in SA	11 pm curfew lifted

SA is a constitutional democracy consisting of national, provincial and local governments. However, the pandemic response was coordinated by the National Coronavirus Command Council (NCCC), a centralized disaster management structure comprising of the president, and ministers and directors general of selected government departments (Steytler *et al*., 2022). Under the DMA, national government could issue alcohol regulations unilaterally and without going through the usual legislative processes (Teer-Tomaselli, 2021). The pandemic therefore constituted a unique situational factor and a ‘crisis’; an extraordinary phenomenon which disrupted and altered the ways in which government and society operates (McConnell, 2011).

Crises present new and urgent public concerns which are often beyond the control of government, and may result in expedited development with major shifts from existing policies (Kingdon and Stano, 1984; Kamkhaji and Radaelli, 2017). According to Grindle and Thomas (1989), the impact of crises includes deviation from established patterns of ‘politics-as-usual’ policy processes, and adaptation and change in the positions, power, and networks of policy actors. Policy conditions may align favourably for a particular issue, forming a ‘policy window’, or strategic opportunity for actors to influence agenda-setting and decision-making processes (Shiffman and Smith, 2007). Crisis episodes can thus shape political priorities and the trajectory of national policy processes (McConnell, 2011).

This study seeks to examine how the COVID-19 crisis influences policy formulation processes for the regulation of alcohol, as part of SA’s pandemic response measures in 2020 and 2021. This includes examining key features of context and the power and position of key actors, and how together, these factors influence policy content in policy formulation processes in a global health crisis context.

The main research question and sub questions are as follows:

How does the COVID-19 crisis, as a key feature of context, influence policy formulation processes for the regulation of alcohol in SA during the period of 30 January 2020 to 31 October 2021?

1. What factors influence policy content in policy formulation processes in a crisis context?
2. How does a global health crisis influence the power and position of key actors involved in and/or affected by the policy formulation process?
3. How does this influence the formulation and reformulation of policies in times of rapid learning and adaptation?

This study contributes to building the field of knowledge on crisis-driven policy formulation. This may also support understandings of the policy context for the advancement of alcohol policy reform in SA and across other LMICs settings, in order to reduce the harms and costs of alcohol to public health and society (Ferreira-Borges *et al*., 2017; Rabiee *et al*., 2017; Bertscher *et al*., 2018; Walls *et al*., 2020; Matzopoulos *et al*., 2020; Rehm *et al*., 2020).

## Conceptual frameworks

A ‘policy cycle’ includes policy identification and issue recognition, policy formulation, policy implementation and policy evaluation (Jenkins-Smith and Sabatier, 1993). This study focuses specifically on policy formulation, which encompasses ‘who is involved in formulating policy, how policies are arrived at, agreed upon, and how they are communicated’ (Buse *et al*., 2012).

**Figure 1.**
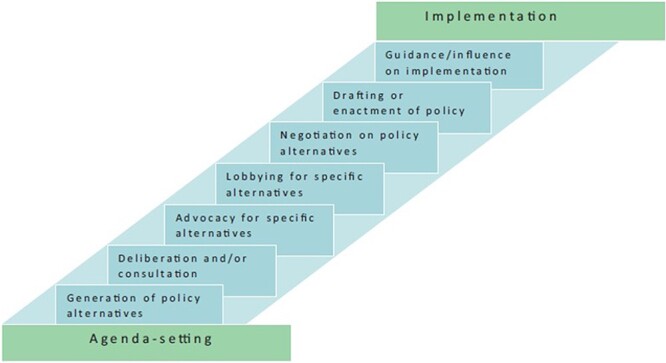
The ‘bits in the middle’ policy formulation process framework by Berlan *et al*. (2014)

The policy formulation framework of Berlan *et al*. (2014) (Figure 1) provided a heuristic through which to identify and analyse activities constituting the policy formulation process. The Berlan *et al*. (2014) framework delineates policy formulation processes into seven ‘bit[s] in the middle’, which fall between agenda-setting and policy implementation. These ‘bits in the middle’ include: (1) generation of policy alternatives, (2) deliberation and/or consultation, (3) advocacy for specific alternatives, (4) lobbying for specific alternatives, (5) negotiation of policy alternatives, (6) drafting or enactment of policy and (7) guidance/influence on implementation (Berlan *et al*., 2014).

The Berlan *et al*. (2014) framework builds on previous conceptualizations, including Sabatier’s stages heuristic (Sabatier, 1991), Grindle and Thomas’s ‘decision-making’ (Grindle and Thomas, 1989) and Kingdon’s streams model of agenda-setting (Kingdon and Stano, 1984), to bring greater analytical clarity to policy formulation (Kingdon and Stano, 1984; Grindle and Thomas, 1989; Sabatier, 1991). The framework provides a structured means through which to identify and analyse various aspects of policy formulation processes, while recognizing the relationship between policy formulation and implementation and how contestation may arise depending on the policy content and actor interests. Although, in the real world, policy formulation processes are non-discrete and non-linear, the framework is a useful heuristic to support investigation (Gilson *et al*., 2018).

The Berlan *et al*. (2014) framework has been tested empirically in health policy analysis research in SA (Bertscher *et al*., 2018). A case study investigation of SA’s 2013 draft Control of Marketing of Alcoholic Beverages bill by Bertscher *et al*. (2018) demonstrates its utility for generation of insights on the formulation of regulatory alcohol policy. However, the framework has not previously been applied to the examination of policy formulation in a crisis context.

**Figure 2.**
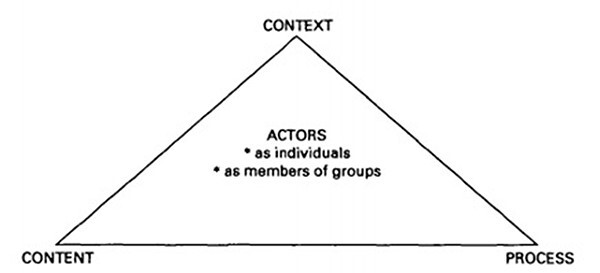
Health policy triangle (Walt and Gilson, 1994)

**Figure 3.**
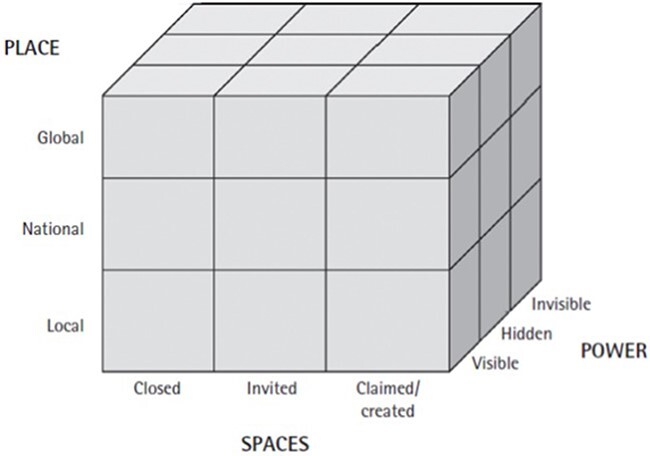
The ‘Power cube’ (Gaventa, 2006)

The study also draws on ideas from the health policy triangle (Walt and Gilson, 1994) (Figure 2), Gaventa’s power cube (Gaventa, 2006) (Figure 3) and Leichter’s categorization of contextual factors (Leichter, 1979) (Table 2) to identify the interconnected concepts of policy content, policy processes, actor interests and their sources of power in the policy process, and the context within which the policy is made (Leichter, 1979; Walt and Gilson, 1994; Gaventa, 2006).

**Table 2.** Leichter’s (1979) categorization of contextual factors

Contextual factors	Description
Cultural factors	Social influences which are often hidden or intangible, such as social norms, values, ideologies and historical aspects
International factors	Transnational inter-dependencies, and the role of global actors and events on policy processes
Structural factors	Permanent or relatively unchanging influences, such as political, legal and economic structures and health system capacity
Situational factors	Idiosyncratic or transient contextual phenomena which influence health policy processes, such as the COVID-19 pandemic

## Methods

### Study design

A single case study design was used, suited to in-depth investigation of complex, non-linear processes and actors within their real-world context (Yin, 2018). This approach allows for flexibility in qualitative data collection and analysis, and application of theory to support analytic generalizability (Yin, 2018).

The case is the policy formulation process for alcohol regulation in SA in the period of 30 January 2020 to 31 December 2021. The time period commences with the WHO’s declaration that the coronavirus outbreak constituted a Public Health Emergency of International Concern (PHEIC) on 30 January 2020, which set in motion SA’s response efforts. The time period ends on 31 December 2021, which marked the end of the lifecycle of and resources for the research project.

### Data collection

Documents formed the primary source of data, and nine in-depth interviews were conducted to triangulate document analysis. The selection criteria for documents included relevance to policy formulation processes for alcohol regulation in SA during the COVID-19 pandemic in 2020 and 2021, or wider historical and contextual relevance to policy processes for alcohol regulation in general. A total of 127 documents were included in the study.^2^ This included publicly available government documents,^3^ documents recommended by interviewees, online and offline media articles and academic literature (see Supplementary data). Documents were sourced using Google, and academic platforms such as Google Scholar, with key search including ‘alcohol ban (South Africa)’, ‘alcohol policy process’, ‘alcohol regulation’, ‘disaster management AND alcohol’, ‘COVID-19 AND alcohol’, ‘alcohol industry AND COVID-19’ and ‘national coronavirus command council AND alcohol’.

Interview participants were purposively selected through a preliminary review of documents and snowball sampling was used to fill emerging gaps until a point of saturation was reached. The main inclusion criterion was that the stakeholder had expert insight and/or experience with the policy formulation processes for alcohol regulation in SA during the COVID-19 pandemic in 2020 and 2021 (Table 3). Participants were sent an email invitation, and semi-structured, hour-long interviews were conducted from March to July 2022. The conceptual frameworks were used to develop an interview guide. Member checking was conducted to improve the accuracy and completeness of interview data. Informed consent for participation and audio recording was obtained, after which interviews were transcribed or field notes were taken. Participants were assured of confidentiality and anonymity.

**Table 3.** Table demonstrating the range of participants interviewed in the study

Actors (political or bureaucratic) who represent the National and Provincial SA government	Actors who represent civil society
Public health officer from a provincial department of health (Interview 1) Member of a provincial alcohol regulatory structure (Interview 4) Public health expert who served in an advisory capacity to government (Interview 9)	Two researchers with expertise in public health and alcohol harms (Interviews 2 and 3) Representative of a civil society alcohol harm reduction advocacy NGO (Interview 5) Public health lawyer (Interview 6) Medical doctor with experience in treating alcohol use disorders and alcohol harm reduction advocacy (Interview 7) Health reporter (Interview 8)

### Data analysis

**Figure 4.**
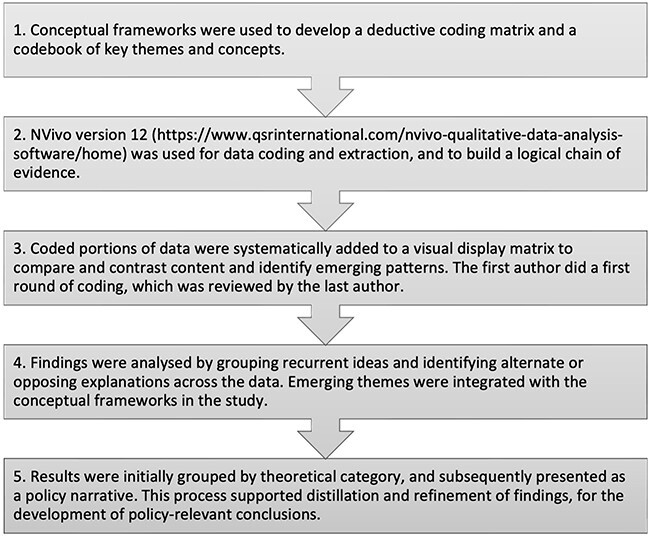
Steps followed for thematic content analysis (Green and Thorogood, 2009; Miles *et al*., 2020; Dalglish *et al*., 2021)

Data were subjected to thematic content analysis, derived from Green and Thorogood (2009) and Miles *et al*. (2020) (Figure 4). Data collection and analysis began with documentary data, and subsequently took place concurrently and iteratively for both documentary and interview data (Miles *et al*., 2020).

Iterative processes of coding and extraction into a visual display matrix were used to integrate the documentary and interview data sets and ‘map’ the key content and topics (Green and Thorogood, 2009). This process allows the researcher to view, and begin to understand the data in its entirety; ‘horizontally’ across the different codes or themes, and ‘vertically’ within each theme (Green and Thorogood, 2009). However, the contributions from each source remain discernible (Kayesa and Shung-King, 2021).

## Results

On 30 January 2020, the WHO announced that the outbreak of a novel coronavirus in the People’s Republic of China constituted a PHEIC (World Health Organization, 2020). The South African government responded rapidly to international public health directives on COVID-19 preparedness and response. On 15 March 2020, just 2 weeks after the first confirmed case of coronavirus in the country, President Cyril Ramaphosa declared a National State of Disaster (Ramaphosa, 2020b).

Initial COVID-19 measures were overseen by the Minister of Cooperative Governance and Traditional Affairs (COGTA), Nkosazana Dlamini-Zuma, and included the closure of schools, limitations on social gatherings, and restrictions on the sale of alcohol (Smart, 2020). Thereafter, a National Coronavirus Command Council (NCCC) was established by the president as the primary decision-making and coordinating body for COVID-19 interventions (Ramaphosa, 2020b). This comprised of the Minister of Health, Dr Zweli Mkhize, and all other cabinet ministers, and was supported by the police service, military and intelligence organizations, and technical experts (Gerber, 2020a).

SA’s initial pandemic response in March 2020 included limitations on alcohol outlet operating times, alongside travel restrictions and social distancing measures (South African Government, 2020b). A week later, the sale, dispensing and transport of alcohol was banned completely, as part of a broader provision to halt all forms of trade and industry deemed ‘non-essential’^4^ (South African Government, 2020a). Individuals found guilty of contravening the alcohol regulations could be fined, arrested, or both, and offending outlets could have their trading licences revoked (Health24, 2020).

The ban on the sale of alcohol was sustained for over 2 months. Thereafter, sales were limited to restricted hours and for off-site consumption only (Department of Co-operative Governance and Traditional Affairs, 2020). However, temporary nationwide bans on alcohol sales were reimposed on 12 July 2020, 28 December 2020 and 28 June 2021, as SA approached the peak of each successive wave of the pandemic (Ramaphosa, 2020c) (Table 1).

In this section, we describe the emergent and often rapid policy formulation processes and discuss key features of context and actor power and positions over time, using the ‘bits’ of the Berlan *et al*. (2014) framework (Figure 1).

### Generating policy alternatives in a time of crisis

The COVID-19 crisis context prompted the generation of alternatives to existing legislation in SA. The origin of the ‘new’ regulations for alcohol was the Disaster Management Act No. 57 of 2002 (DMA), which explicitly allows for the ‘suspension or limiting of the sale, dispensing or transportation of alcoholic beverages’ during a national state of disaster (Disaster Management Act, No. 57 of 2002, 2002).

SA was one of only a few countries to ban alcohol sales completely at points in time during the pandemic, a decision that preceded the WHO’s recommendation on regulating alcohol access in the crisis context (Matzopoulos *et al*., 2020; World Health Organization, 2020). Other countries that introduced temporary alcohol sales bans include Botswana, India, Greenland and parts of Canada (Maker, 2020; Maphisa and Mosarwane, 2022). On the contrary, countries including Australia, China, Germany, the UK and USA introduced measures such as off-premises online alcohol sales to enable access to alcohol during the crisis (Keric and Stafford, 2021; Stockwell *et al*., 2021).

In explaining the lockdown measures, President Ramaphosa emphasized the need to ‘ensure that hospitals are not overwhelmed’, as had been observed in other countries (Ramaphosa, 2020c). Urgency to protect hospital capacity was heightened by local resource constraints and population vulnerabilities, including a high prevalence of HIV, TB, poverty and malnutrition (Ramaphosa, 2020a; 2021a). The Police Minister also highlighted health system challenges in his public comments at the time (Health24, 2020).

*‘For 21 days, please stay sober,’ said police minister Bheki Cele at a media briefing. He said that the expected decline in accidents and assaults due to the ban on buying alcohol will free up much-needed space in hospitals during the coronavirus crisis* (Health24, 2020).

During the pandemic, emergency responders shared anecdotal evidence linking reduced alcohol availability to reduced trauma caseloads (Kotze, 2020). However, amidst emerging contestation from the alcohol industry, public health experts anticipated the need for more robust evidence to support alcohol policy alternatives (Kotze, 2020; Matzopoulos *et al*., 2020). Researchers at provincial health authorities, academic institutions, health facilities and the South African Medical Research Council (SAMRC), a para-statal medical research organization, played a proactive role in highlighting pre-existing evidence on alcohol-related trauma, as well as data emerging in real-time from national mortality data bases and hospitals^5^ (Zsilavecz *et al*., 2020; Moultrie *et al*., 2021; Navsaria *et al*., 2021; Chu *et al*., 2022).

The generation of scientific evidence played a significant role in developing alcohol policy alternatives. For example, in the midst of an exponential rise in COVID-19 case numbers in July 2020, technical experts were invited to advise a subcommittee of the Ministerial Advisory Committee on COVID-19 on the projected health system impacts of different policy options (Table 4) (Mitchley, 2020a). The evidence compiled by the working group was taken forward to the Minister of Health and the NCCC, and featured in government communications ahead of the decision to reimpose a complete ban on alcohol sales on 12 July 2020^6^ (Interview 3, 2022).

**Table 4.** Alcohol policy alternatives and recommendations for the crisis context generated by the Technical Working Group on Alcohol in July 2020 (Technical Working Group on Alcohol, 2020)

Alcohol policy alternative	Recommendations and rationale
Re-imposing a complete ban on the sale of alcohol	It was estimated that a ban could result in a reduction of 20% of all trauma admissions/40% of alcohol-related trauma presentations by the end of the third week. This was projected to reduce the burden on healthcare workers and the demand for hospital beds, ICU facilities and ventilation capacity in the crisis context.
Using a combination of less restrictive evidence-based strategies to limit alcohol availability, drink driving countermeasures and policing of illegal drinking, and restrictions on advertising and packaging	These alternatives were projected to have a lesser impact on trauma rates, and to be more difficult to implement.
Using a differentiated approach, whereby provincial or local government authorities determine alcohol regulations	Despite the variation in COVID-19 caseloads across provinces, a national level response was recommended due to the logistics of controlling the distribution of alcohol across provincial borders.
Other recommendations	Government should maintain transparency and share the rationale and evidence behind alcohol regulations with the public. Government communications should provide details on where and how to seek help for alcohol withdrawal or dependence. Trauma related to alcohol should be made notifiable to support the generation of evidence on health and economic impacts.

The Ministerial Advisory Committee did not always provide specific recommendations regarding alcohol regulation. However, they regularly provided evidence of COVID-19 infection rates and the projected threat to hospital capacity. This was submitted to government and played a key role in decision-making^7^ (Mkhize, 2020a; Mashego, 2021a).

Temporary alcohol sales bans in December 2020 and June 2021 were supported by growing evidence demonstrating the effectiveness of this approach in relieving the health system during periods of high COVID-19 case numbers (Zsilavecz *et al*., 2020; Moultrie *et al*., 2021; Navsaria *et al*., 2021).

During the crisis, alcohol industry stakeholders contested public health evidence to promote deregulation of alcohol (Interviews 2 and 3, 2022). Alcohol industry-funded research reports argued, firstly, that there was insufficient evidence to support the alcohol regulations, emphasizing the effects of concurrent curfew regulations, and, secondly, that alcohol bans were futile due to illicit alcohol trade (Mitchley, 2020b; Planting, 2021; Tshikalange, 2021). Industry-linked researchers also attempted to discredit pre-existing public health evidence through commentary in a prominent academic medical journal (Murray and Barr, 2022). Public health experts challenged these commentaries and other reports for their methodological limitations, a lack of peer-review and conflicts of interest due to industry funding (Matzopoulos *et al*., 2022; Mitchley, 2020a; Planting, 2021; Tomlinson, 2021). However, the media continued to disseminate alcohol industry reports unquestioningly as evidence of ‘major flaws in South Africa’s lockdown alcohol bans’ (BusinessTech, 2021b).

### Processes of deliberation, consultation, lobbying and negotiation in times of crisis

This section covers three ‘bits’ of the policy formulation process: (1) ‘deliberation and/or consultation’; (2) ‘lobbying for specific alternatives’; and (3) ‘negotiation for specific alternatives’^8^ (Berlan *et al*., 2014). These activities were characterized by shifting configurations of actor power and reduced stakeholder access to national decision-making spaces. Positions on alcohol within groups of stakeholders were not homogenous, and the evolving nature of the crisis and policy content resulted in a dynamic spectrum of perspectives. However, actors may be understood broadly to be driven by different interests including public health, economic or commercial interests.

Early in the pandemic, the decision-making space was explicitly ‘closed’ (Gaventa, 2006). Policy formulation processes were confined to a relatively small group of high-level government officials through the establishment of the NCCC. Under the DMA, the decision to impose a national lockdown did not involve the usual stages of deliberation and consultation with the National Assembly, provincial and local government actors, and the public (Lukani, 2020; Singh, 2020). Civil society stakeholders, including prominent thought leaders, journalists and the public, questioned the power of the NCCC, and criticized the top-down approach to policymaking^9^ (Haffajee, 2020; Reddy *et al*., 2020). In relation to alcohol policy, participants in this study indicated that stakeholders lacked opportunities for participation and were ‘caught unaware’ by the initial regulations (Interviews 2, 3, 4 and 5, 2022).

Over the course of the pandemic, selected stakeholders were afforded access to the national policy arena through ‘invited spaces’ for participation (Gaventa, 2006). However, these spaces did not confer a high degree of influence on decision-makers or alcohol policy content. For example, while subnational government actors regularly engaged with national government via the President’s Coordinating Council during the crisis, anecdotal evidence suggests that meetings served as a ‘conduit for information and instructions rather than a platform for negotiation’ (Steytler *et al*., 2022). Furthermore, domains of alcohol regulation that were previously under provincial and municipal authority shifted entirely to the national level under the DMA. Within the altered legislative context, some provincial actors turned to lobbying to promote local level health and economic priorities, and political party interests^10^ (Meyer, 2021; Ngqangashe *et al*., 2021).

Selected clinicians, scientists, and researchers were invited to advise the Minister of Health on the pandemic response through three Ministerial Advisory Committees (MACs).^11^ The establishment of the MACs supported government assurances to the public that response measures were guided by ‘the advice of scientists’ (Ramaphosa, 2020d). However, as an advisory body, the MACs’ level of influence was explicitly bounded and the NCCC retained the final say on policy decisions (Mkhize, 2020a). MAC reports and recommendations were initially withheld from the public, limiting broader stakeholder engagement (Richter *et al*., 2022).

In some instances, alcohol industry stakeholders were invited to make submissions ahead of new regulations being gazetted, or participate via the National Economic Development and Labour Council (NEDLAC) (Ramaphosa, 2020d). NEDLAC is a formal state platform for businesses and labour organizations to engage with government on trade policy agendas and decisions (Milsom *et al*., 2022). NEDLAC does not officially include the National Department of Health and is perceived as difficult to access by civil society organizations (Milsom *et al*., 2022). However, during the pandemic, the space was expanded to include public health representatives and civil society groups advocating alcohol harm reduction (Interviews 1, 2, 5 and 6, 2022). It is unclear to what extent negotiations and lobbying through NEDLAC influenced policy decisions. One participant in this study suggested that the ‘tug-of-war’ between invited stakeholders may have had a balancing effect in ensuring that regulations were instituted reasonably, and according to the fluctuating severity of the health crisis (Interview 1, 2022).

*Everybody was deeply frustrated. We don’t know how much influence all of us had, compared to how much influence the liquor industry had. Because the liquor industry was also complaining and saying that government was not listening to them* (Interview 5, 2022).

Civil society stakeholders sought to increase their level of influence through coalition building and lobbying. Alcohol industry activities were largely coordinated by pre-established representative organizations, encompassing stakeholders across the local and international supply chain of growers, manufacturers, distributors and retailers (Ngqangashe *et al*., 2021). These organizations ‘claimed’ space in the policy arena by lobbying for de-regulation^12^ and self-regulation though industry-funded initiatives^13^ (Cele *et al*., 2020; SABC, 2020; BusinessTech, 2020a; 2020c; Kahla, 2021). Alcohol industry actors were not homogeneous in their policy positions and strategies over time. Internationally linked alcohol retailers, such as South African Breweries (SAB), used financial and litigation threats and penalties to gain access to decision-makers and leverage in negotiations (Areff, 2021; Planting, 2021; Smith, 2021). The head of trade and economics of the European Union delegation to SA reportedly offered support to major alcohol industry groups for ‘activities to avoid/mitigate other bans’ (Movendi International, 2021). This demonstrates Gaventa’s (2006) concept of ‘hidden’ power dynamics, which operate to amplify and increase the legitimacy of certain issues and voices in the policy process.

While powerful spaces for civil society participation were ‘created’ during the pandemic,^14^ they were not focused on the issue of alcohol regulation (C19 People’s Coalition, 2021; van Ryneveld *et al*., 2020). Civil society stakeholders had limited resources to draw on, and a participant in this study noted that the potential for grassroots mobilization was limited by social distancing regulations (Interview 7, 2022). Nevertheless, organizations with a long-standing focus on alcohol harm reduction actively lobbied for evidence-based public health-oriented policy alternatives, for both the pandemic context and beyond^15^ (Fokazi, 2020; George Herald, 2020). Building coalitions with public health experts and allied interest groups (e.g., organizations concerned with child welfare, gender-based violence, road safety and gun control) strengthened lobbying efforts, and proved useful in gaining some access to national level spaces (Interview 5, 2022).

### Advocacy for specific policy alternatives in the crisis context

Advocacy for specific alternatives involves stakeholder efforts to ‘advance their particular policy options’ by targeting the bureaucracy and the broader public (Berlan *et al*., 2014).

#### Government

Communications from the presidency and national Department of Health to the public conveyed the urgency of protecting health system capacity, along with supporting evidence behind the alcohol regulations, particularly during periods of high COVID-19 hospital admissions (Mitchley, 2020a; Ramaphosa, 2020a,c; Kgosana, 2021; Ngqangashe *et al*., 2021; Steytler *et al*., 2022).

*At a time like this, when every bed, every healthcare worker, every ounce of oxygen is needed, it would be unforgivable to identify a clear burden on the healthcare system and do nothing about it. – Health Minister, Dr Zweli Mkhize* (Mitchley, 2020a).

A survey conducted across a representative sample of over 4000 SAs in June 2020 found that most respondents (63%) believed that alcohol bans were important (News24, 2020). However, state messaging on alcohol was weakened by a lack of cohesion across sectors^16^ (Haffajee, 2020; Ngqangashe *et al*., 2021).

#### Civil society actors

Public health experts, healthcare workers and alcohol-related interest groups gained elevated media attention during the pandemic (SABC News, 2020; Ngqangashe *et al*., 2021). Media platforms, including social media, were used to raise public awareness of the magnitude of the health and social harms resulting from alcohol consumption, and trauma in particular (Kotze, 2020; Bartlett *et al*., 2023).

Several participants referred to New Year’s morning in 2021, when images and videos of empty hospital trauma centres were shared widely on social media and popular news media platforms (Interviews 1, 6 and 7, 2022). Within the context of high rates of COVID-19 hospital admissions, President Ramaphosa announced an extension of the third alcohol ban soon after to ‘protect our health services at this crucial time’ (Ramaphosa, 2021a).

*There’s that famous photo of Chris Hani Baragwanath [hospital] on the 1st of January 2021 – an empty emergency centre. I mean, this is unheard of… Within the profession, but also outside, I think there was more of an awareness that alcohol plays a huge role in terms of the burden on our health system*(Interview 7, 2022).

Within the context of prolonged periods of complete alcohol sales restriction, both proponents and opponents of the alcohol regulations highlighted the plight of persons struggling with alcohol dependence or withdrawal (Myers *et al*., 2010; Matzopoulos *et al*., 2020; Qukula, 2020; Gerber, 2020b). While the issue was briefly acknowledged by the president in June 2020, interventions to improve SA’s scarce alcohol treatment and counselling services did not feature in the disaster response and represented a missed public health opportunity (Gerber, 2020b; Qukula, 2020; Ramaphosa, 2020c).

#### The alcohol industry

Alcohol industry advocacy efforts were strategically adapted to the evolving crisis context. At the start of the pandemic, and during periods of increased COVID-19 cases, industry actors positioned themselves as working in support of national disaster response efforts by highlighting their social responsibility efforts in the COVID-19 response (SABC, 2020; BusinessTech, 2020b) and the plight of economically vulnerable stakeholders, such as independent tavern owners (Ngqangashe *et al*., 2021), while emphasizing the broader contributions of the alcohol industry to the economy. As the sense of urgency associated with the health crisis waned and pandemic ‘fatigue’ set in, alcohol industry messaging became increasingly alarmist, and openly critical of the evidence and of government (Abdool Karim & Jacobs, 2020; Kahla, 2021; Maduray, 2021; Ngqangashe *et al*., 2021).

In July 2021, a wave of civil unrest occurred in two provinces, sparked by political contestation and fuelled by worsening unemployment and socio-economic inequality. The economic impacts of the unrest and allegations of corruption, followed by resignation of the Minister of Health, were incorporated into the alcohol industry narrative that alcohol bans were harmful and unjustified (TimesLive, 2021; Mashego, 2021b). In the run-up to national municipal elections in November 2021, members of the main political opposition party suggested that the alcohol industry was being used as a scapegoat for deficiencies in the disaster response (Macpherson, 2021). Within the context of rising political and economic pressures, and lower numbers of hospitalizations anticipated for the Omicron variant, an alcohol ban was not imposed ahead of the fourth wave of the pandemic in December 2021 (Business Tech, 2021c). On the contrary, licensed alcohol outlets were permitted to revert back to pre-pandemic operating conditions (Ramaphosa, 2021b).

#### The media

During the pandemic, stakeholders drew on the ‘invisible power’ of the media to shape public perception (Gaventa, 2006). A participant in this study who works for an online newspaper suggested that media actors afforded industry stakeholders an undue degree of attention and credibility during the pandemic (Interview 8, 2022). This included publishing of industry-funded research reports and press releases from industry representative organizations, without seeking alternate stakeholder perspectives (Kahla, 2021; BusinessTech, 2021b).

*It wasn’t a balanced picture, but that is largely because they [the media] were being fed so much material from the industry …* (Interview 4, 2022).

Government and civil society stakeholders contested the alcohol industry’s paid use of the media on several occasions. In January 2021, the Department of Health criticized SAB for enlisting local celebrities to oppose alcohol regulations on social media, without declaring that the content was part of a funded campaign (Sifile, 2021). A second example involved alcohol industry funded advertorials which appeared in the *Sunday Times*, SA’s biggest Sunday newspaper, in February 2021 (Govender *et al*., 2021). Following complaints by the Southern African Alcohol Policy Alliance (SAAPA), the Press Ombud found that there was an ‘unacceptable blurring of the lines’ between editorial content and sponsored content, and the *Sunday Times* was required to apologize to its readers (Govender *et al*., 2021).

### Drafting and enactment of policy in the crisis context

Under the state of disaster, the Minister of COGTA^17^ was responsible for drafting disaster regulations in collaboration with other cabinet ministers (Department of Co-operative Governance and Traditional Affairs, 2020). The centralized decision-making context enabled rapid, overlapping cycles of drafting and re-drafting of alcohol regulations.

*I am the one who signs off because someone has to take responsibility for the regulations, but I am in no way in charge and making decisions alone*—Minister of COGTA (Felix, 2020).

### Guiding or influencing implementation in the crisis context

The final ‘bit’ is defined as ‘activities that continue to shape the content of policy after legislation’ (Berlan *et al*., 2014). Given fast-paced policy cycles with limited opportunities for participation, stakeholders turned to the SA constitution to contest enacted alcohol regulations. The national state of disaster, unlike a state of emergency, retained constitutional rights to protest or challenge policies in court (Abdool Karim and Kruger, 2021). DMA regulations state that any incursions on human rights must be reasonable and justifiable in relation to the expected public health benefit, and consistent with constitutional values of dignity, freedom and equality (Abdool Karim and Kruger, 2021), and this formed the rationale for litigation (Table 5) (Gerber, 2020b; Heiberg and Char, 2021; News24, 2021).

**Table 5.** Key examples of litigation threats and court challenges relating to alcohol regulation in SA during the COVID-19 pandemic in 2020 and 2021

Date	Actor	Argument	Outcome	References
April 2020	Gauteng Liquor Forum (representing over 20 000 taverns and shebeens) threatened a constitutional court challenge but did not follow through.	Argued that the initial alcohol ban violated the right to freely choose one’s trade, occupation or profession.	Request to resume alcohol sales was declined by the Presidency in April 2020.	(SABC News, 2020; The Presidency of SA, 2020)
Jan–Dec 2021	Vinpro (representing 3500 wine producers), supported by the Western Cape provincial government, approached the Western Cape High Court.	Argued for alcohol regulations to be decided upon at provincial level, based on differentiated COVID-19 risks.	Application dismissed.	(BusinessTech, 2021b; Heiberg and Char, 2021; Smith, 2021)
Jun 2021–Jan 2022	SAB approached the Western Cape High Court.	Argued that there was inadequate consultation and parliamentary scrutiny, and that COGTA Minister had no power to suspend the sale and distribution of alcohol.	Urgent application dismissed with costs. SAB denied leave to appeal by the Supreme Court in Jan 2022.	(News24, 2021; Planting, 2021; Smith, 2022)

However, efforts to influence implementation were limited by the overriding public health priorities of the crisis context. In July 2020, protests against the alcohol regulations by hospitality stakeholders were dispersed by the police, since gatherings were deemed unsafe and illegal under the DMA (Hendricks and Postman, 2020). Slow-moving litigation processes were also ineffectual in altering the course of alcohol policy decisions, given the rapid pace at which alcohol regulations were modified (Smith, 2021; BusinessTech, 2021a; Steytler *et al*., 2022). Court responses aligned with public health evidence and demonstrated understanding of the challenging decision-making context of the crisis^18^ (Abdool Karim and Kruger, 2021; News24, 2021).

*‘Given the circumstances and the limited timeframe in which the Minister had to act, it cannot be said that she acted in a procedurally unfair manner,’ the judge said* (News24, 2021).

## Discussion

Recognizing the COVID-19 pandemic as a unique situational factor, key factors that influenced policy formulation in the SA crisis context in 2020 and 2021 included the: (1) perceived urgency of the crisis, (2) centralization of power, (3) competing economic and public health values and (4) the availability of historical and contemporary evidence.

### Perceptions of urgency

Policy formulation processes and content for the pandemic response were initially driven by a strong sense of urgency among stakeholders in government, the electorate and the mass media. This contained both an objective measurable element of risk, derived from international COVID-19 experiences and evidence, and a socially constructed element of how risks were understood within the context of health system resource constraints and population health vulnerabilities. The sense of crisis enabled a radical acceleration in decision-making processes. Factors that were understood to exacerbate the crisis, such as alcohol, gained a particularly high degree of receptivity to policy change. This prompted a shift from incrementalism to innovation in alcohol policy content, followed by regular readjustments as the perceived pandemic urgency fluctuated over time.

The importance of perceived urgency in this case study is consistent with empirical research on COVID-19 responses locally and globally (Amri and Logan, 2021; Schneider *et al*., 2021; Steytler *et al*., 2022) and with previous respiratory disease outbreaks of Severe Atypical Respiratory Syndrome (SARS) in 2003, the H1N1 flu in 2009, and the Middle East Respiratory Syndrome (MERS) in 2012 (Fritzen, 2004; Smith, 2006; Lawson and Xu, 2007; Fineberg, 2014; Lee and Jung, 2019). Lawson and Xu (2007) describe ‘fear itself’ as a policy instrument that facilitated faster state control during the SARS outbreak in 2003. Building on the work of Grindle and Thomas (1989), Fritzen (2004) posit that ‘not all crises are created equal’, and suggest that the greater the degree of the perceived threat to socio-economic and political stability, the more likely that policy formulation processes and outcomes are significantly different from the status quo.

### Centralization of power

International responses to the COVID-19 pandemic involved redistributions of power in decision-making processes both ‘within’ government and ‘between’ national and subnational levels of government. Most countries, including SA, demonstrated a trend towards centralization (Steytler *et al*., 2022). This enabled rapid cycles of policy formulation and implementation during the crisis. The positions taken by policy elites on the temporary regulation of alcohol were shaped by their understanding of the link between alcohol availability and health system capacity.

Within the context of centralized power, non-state policy actors developed coalitions and attempted to ‘claim’ or ‘create’ alternative decision-making settings to advance their policy goals and serve their organizational needs (Gaventa, 2006; Weible and Nohrstedt, 2012). This is consistent with the theory of ‘venue shopping’, which suggest that choices of decision-making settings are experimental and shaped by new understandings of the nature of the problem and the policy environment (Baumgartner and Jones, 1993; Pralle, 2003).

Normally, alcohol industry stakeholders gain privileged access to the institutional policy arena and can intervene when draft bills are debated in parliament (Parry, 2010; Bertscher *et al*., 2018). However, with compressed policy cycles and closed decision-making spaces, the alcohol industry was compelled to rely on lobbying and advocacy in the public domain as a strategy to influence policy processes. This is consistent with international reports of persistent and coordinated alcohol industry lobbying and advocacy efforts to widen alcohol availability during the pandemic (Keric and Stafford, 2021; Movendi International, 2021; Stockwell *et al*., 2021). Furthermore, it reflects the general degree of responsiveness and involvement of alcohol industry actors in public health policymaking to advance commercial interests, outside of the crisis context (Moodie *et al*., 2013; Savell *et al*., 2016; Ferreira-Borges *et al*., 2017; McCambridge *et al*., 2019).

Public health experts and civil society groups also responded to the shift in the policy environment, although with fewer resources, and mobilized to generate political momentum for alcohol policy through coalition building, research, advocacy and contestation of alcohol industry claims. These strategies have also been observed outside of the crisis context (Lesch and McCambridge, 2021; Gage *et al*., 2024).

Lawson and Xu (2007) have argued that the power of scientific experts typically increases during health emergencies, and this may extend post-crisis, with a reduction in the normal institutional separations of scientific and political power (Lawson and Xu, 2007). Empirical research on the COVID-19 pandemic suggests that in many countries, the roles and boundaries of power of scientific experts on government advisory committees were not clearly defined (Colman *et al*., 2021; Morales, 2021). In SA, while public health experts and civil society groups enabled government to better resist alcohol industry pressures for a period during the COVID-19 pandemic through research, advocacy and participation in government advisory bodies (Morojele *et al*., 2021), their role and influence were explicitly limited, and decision-making remained an exclusive state capacity.

### Competing priorities

Countries globally faced the dilemma of trying to maintain economic stability while protecting public health, demanding choices as to how health and social costs and benefits are valued and re-negotiated over time in crisis-driven policy formulation processes (Weible *et al*., 2020). SA initially appeared to take a strong stance in favour of public health objectives, by instituting one of the strictest lockdowns globally. The alcohol regulations were rationalized through references to public health values and evidence. Institutional spaces for non-state actor participation were expanded beyond bureaucratic norms to include public health experts, in stark contrast to prior alcohol policy processes, which saw economic and commercial interests dominate deliberations on state platforms (Parry, 2010; Bertscher *et al*., 2018).

However, the implication that public health was prioritized over the economy during the pandemic is not totally accurate. The alcohol regulations were explicitly presented as exceptional and, most importantly, temporary threats to commercial activity. The regulations were not an alcohol harm reduction strategy, but rather a COVID-19 harm reduction policy, and did not necessarily signify a stronger long-term political stance on alcohol harms.

This aligns with empirical research on responses to the SARS outbreak in 2003, in which governments imposed temporary travel restrictions; firstly, to minimize political consequences of neglecting public health imperatives, and secondly, to facilitate a swift return to normal trade (Fritzen, 2004; Lawson and Xu, 2007). This suggests that while public health values are used to legitimize crisis-driven decision-making in the short-term, they do not change the political considerations and normative economic orientations that underlie policy-making (Lawson and Xu, 2007; Weible *et al*., 2020). In unitary policy sub-systems, dominant policy actors may reframe the problem once the crisis has passed, and tone down the policy implications that may emerge from the crisis as a means of restoring the status quo (Nohrstedt and Weible, 2010).

Nonetheless, the regulations highlighted the extent of societal harms from alcohol consumption, offering a window into a different policy narrative. Depending on how public health learnings and values are taken up by actors, the COVID-19 regulations may well influence the long-term trajectory of alcohol policy processes.

### The role of evidence

The initial months of the COVID-19 pandemic were characterized by a high degree of uncertainty, requiring policy learning and adaptation (Weible *et al*., 2020; Zaki and Wayenberg, 2021). While SA drew lessons on COVID-19 from international public health responses, the idea of banning alcohol sales did not originate from other countries. On the contrary, several governments deemed alcohol essential during the crisis due to concerns around alcohol withdrawal, losing popular support, economic impacts and alcohol industry pressures (Keric and Stafford, 2021; Stockwell *et al*., 2021).

SA’s alcohol regulations originated though policymakers drawing on their own pre-existing knowledge of the link between alcohol availability and health system capacity, and epistemic policy learning from a proactive network of public health and alcohol experts. Both pre-existing and emerging scientific evidence on alcohol-related trauma featured prominently in deliberations across state and non-state platforms, supported by anecdotal evidence and experiential learning over the course of the pandemic.

The COVID-19 moment opened new ways of thinking about the relative balance of public interest and private profit. The consequences of the ban, which was an ‘experiment’ that could never have been implemented under ‘normal’ circumstances, were powerful in exposing an underlying reality previously hidden from the policy agenda.

While evidence played a significant role in supporting public health-oriented alcohol regulations, it is important to note a few limitations. Firstly, understandings of what constitutes ‘evidence’ vary widely (Stern, 1997; Zaki and Wayenberg, 2021). State actors and the media may weight research findings equally, regardless of the quality of scientific inquiry, sources of funding and conflicts of interest. Consistent with Bertscher *et al*. (2018), this created a ‘battle for evidence’ during the crisis, with the alcohol industry investing heavily in strategies to produce a counter-narrative to public health evidence (Mitchley, 2020b; Planting, 2021; Tomlinson, 2021; Matzopoulos *et al*., 2022). As a result, actors may draw widely different lessons from the same experiences.

Secondly, policy learning does not necessarily lead to on-going refinement of policy solutions. Following the initial alcohol ban and policy learning around its proven impact on trauma admissions, decision-makers adopted a patterned or cyclical alcohol policy response to subsequent COVID-19 surges. This reflected a ‘policy core belief’ on what causes the policy problem of hospital bed shortages and how a government can solve it (Weible and Nohrstedt, 2012). This is consistent with the concept of ‘bounded rationality’, as described by Grindle and Thomas (1989), and has particular relevance for policy learning in infectious disease outbreaks and other crisis contexts with a fluctuating or recurrent nature.

Lastly, the ideal of ‘evidence-based policymaking’ is constrained by the politicization of science. Linking alcohol regulations to experts and evidence serves to legitimize policy choices, by signalling to the public and to the courts that decisions were rational and informed (Lawson and Xu, 2007; Weible *et al*., 2020). Yet, concurrently, political considerations may remain the predominant influence on decision making (Fritzen, 2004; Lawson and Xu, 2007; Zaki and Wayenberg, 2021). This has implications for the post-crisis period for alcohol regulation in SA, in which important lessons may be overlooked due to lack of fit with prevailing contextual priorities, mindsets and power structures.

## The applicability of the frameworks used

In this study, the Berlan *et al*. (2014) framework was found to be a useful heuristic for identifying the events, activities and processes that constitute the ‘policy formulation’ stage of the policy cycle within a crisis context. However, as noted by Bertscher, et al., 2018, given the non-discrete and non-linear nature of policy processes, there was considerable overlap between the bits. While Berlan *et al*. (2014) do acknowledge the policy process as complex, the crisis context, which includes rapid recurring policy cycles, adds further complexity to the policy formulation process.

Because of this, we have explicitly introduced the concepts of time available for decision-making and perceived urgency as important features to consider in policy processes. This introduces the importance of temporality and increasing non-linearity in the understanding of policy formulation. We suggest placing time and perceived urgency, as additional concepts, beneath the Berlan *et al*. (2014) framework between agenda-setting and implementation, to illustrate that they are enduring aspects in the policy formulation process that must be considered.

## Limitations

Uncovering all the overlapping policy formulation processes taking place during the study period was not possible. While this study provides a snapshot of the dominant actors and events, and includes multiple documentary sources, alcohol industry informants and actors absent from mainstream media were excluded. Furthermore, some interviewees struggled to recall details and the exact chronology of events. In complex real-world research, it is not possible to uncover the full extent of existing information. To address the limitations of a bounded data set, we were guided by theory and public health perspectives to derive analytical generalizations.

## Practical implications for alcohol policy formulation in the future

This case study demonstrates that it is possible in practice to put in place alcohol regulations for the benefit of the public’s health. While this occurred specifically within a crisis context and was linked to COVID-19 response measures, the experience illustrates that under particular circumstances, it is possible for evidence to outcompete political and commercial influence in alcohol policy formation.

The alcohol policy environment in SA is characterized by political contestation around ‘evidence’ (Bertscher *et al*., 2018). However public awareness of the harms that could be prevented by stronger alcohol regulation is now widespread, given the almost incontrovertible links that emerged between open access to alcohol and traumatic injury during COVID-19 (Moultrie *et al*., 2021; van Hoving *et al*., 2021; Chu *et al*., 2022). Anecdotal evidence, such as the images of an empty trauma unit at Chris Hani Baragwanath Hospital shared by healthcare workers on New Year’s morning in 2021 (Mitchley, 2021), amplified the impact of public health evidence. This heightened public awareness will be difficult to ‘spool back’ in future engagements and raises insights for policy formulation processes for the regulation of alcohol more generally.

Given the key role of existing and emerging evidence in decision-making for alcohol regulation during the crisis, we encourage continued support of public health research on the harms and costs associated with alcohol, particularly in relation to the effects on health system capacity and resources. Considering the financial power of the alcohol industry to shape perceptions around alcohol regulation through paid use of the media, for example, industry-sponsored social media campaigns and advertorials (Govender *et al*., 2021; Sifile, 2021), knowledge translation and dissemination of public health evidence in accessible formats is crucial to challenge alcohol industry narratives. This could also raise the consciousness and understanding of both the public and decision-makers, and encourage widespread support for public health efforts to reduce alcohol harms and costs.

## Conclusion

The study demonstrates the utility of the Berlan *et al*. (2014) framework and also shows the importance of embedding this within the health policy triangle (Walt and Gilson, 1994) to investigate the intersection of contextual factors, actor power and policy content within policy formulation processes (Walt and Gilson, 1994).

This study illustrates that (1) consistent with international literature, perceptions of urgency of the health crisis influence the degree to which policy processes and content are transformed, and in the case of SA’s alcohol regulations, may draw attention to previously neglected preventable disease burdens; (2) centralization of power in crisis contexts enables rapid and decisive cycles of policy formulation but limits stakeholder participation, resulting in the mobilization of actor networks with diverse strategies to influence policy processes through the public arena; (3) actors may use different forms of evidence (ranging from scientific research to marketing spin) to support competing agendas in crisis contexts, pointing to the need to address and manage conflicts of interest in scientific and media platforms, including disclosure of alcohol industry funding; and (4) epistemic and experiential policy learning and adaptation plays a key role in policy formulation processes at the onset of a crisis, but is less influential over time within the context of competing economic and political priorities. The process does, however, open a window that can possibly influence the future policy trajectory on alcohol harm reduction.

## Supplementary Material

czae055_Supp

## Data Availability

The documentary data underlying this article are available in the article and in its online supplementary material. The interview data underlying this article cannot be shared publicly to maintain the privacy of the individuals who participated in the study.

## Footnotes

1. Under the national state of disaster in SA, lockdown regulations involved varying degrees of restriction on trade, movement and social gathering. A ‘risk-adjusted approach’ was adopted to enable responsiveness to fluctuating COVID-19 infection rates and health system demand. This involved a five-level alert system, with levels 5 to 1 representing stepwise relaxation of restrictions (Dlamini Zuma, 2020).

2. The majority of these documents are dated between January 2020 and December 2021. Some documents that pre-dated (*n* = 5) or post-dated (*n* = 6) the study period were included for relevance to understanding the context for alcohol regulation in SA.

3. Records of government documents, such as memos, speeches and press releases, are publicly accessible on the South African government’s official website (https://www.gov.za) and the official coronavirus (COVID-19) online news and information portal (https://sacoronavirus.co.za).

4. National lockdown regulations also included a highly controversial ban on the sale of tobacco for over 4 months. The rationales and strength of evidence behind the alcohol and tobacco bans were distinctly different; however, the issues were often conflated in media reports (Cele *et al*., 2020; Zulu, 2020; Hunter, 2020).

5. Data from the recently established electronic health record registry (HECTIS) in the Western Cape province feature in the Sentinel Trauma Report compiled by the Western Cape Department of Health in July 2020, a report produced by the Technical Working Group on Alcohol in July 2020, and a study published in the South African Medical Journal in February 2021 (Hyman, 2020; Technical Working Group on Alcohol, 2020; Navsaria *et al*., 2021).

6. Evidence from the technical working group report was cited in official communications to the media and public from the president, Minister of Health and Department of Health (Department of Health, 2020; Mkhize, 2020b; Ramaphosa, 2020a; SA Department of Health, 2020).

7. On 23 December 2021, a memo from the Ministerial Advisory Committee to the Health Minister reported that a rapid increase in the number of COVID-19 cases presented a serious threat to hospital capacity. The memo did not offer a specific recommendation for the regulation of alcohol; however, a complete restriction on the sale of alcohol was announced soon after, and was sustained from 28 December 2020 to 1 February 2021.

8. ‘Deliberation and/or consultation’ entails government discussing policy alternatives with those internal and external to the government. ‘Lobbying for specific alternatives’ involves activities with the intention of directly or indirectly influencing decision-makers. ‘Negotiation for specific alternatives’ involves stakeholder negotiations with government, with the aim of influencing specific details of the policy content (Berlan *et al*., 2014).

9. On 27 April 2020, two advocates with long-standing public administration experience penned an open letter to the Presidency, raising concerns about the constitutionality of a centralized decision-making body (Haffajee, 2020). Research undertaken using email responses received by government from the public in July 2020 indicated dissatisfaction with the top-down approach to the disaster response (Reddy *et al*., 2020).

10. The Western Cape province, led by the main opposition party, the Democratic Alliance, was particularly active in lobbying national government. In many instances, the province argued in support of de-regulation, citing concerns for the local wine sector and economy (Hyman, 2020; Meyer, 2021).

11. The MAC on COVID-19, the Vaccine MAC and the Social Behavioural Change MAC.

12. When a complete ban was in place, alcohol stakeholders lobbied government for deregulation in the form of time-based restrictions. When time-based restrictions were in place, alcohol stakeholders lobbied government for extended trading hours (BusinessTech, 2020a; 2020b; Kahla, 2021; Ngqangashe *et al*., 2021).

13. Alcohol industry initiatives for self-regulation included measures to improve COVID-19 safety at alcohol outlets, encouraging outlet compliance with licensing conditions, and Corporate Social Responsibility campaigns to promote ‘responsible drinking’ (Cele *et al*., 2020; SABC, 2020; BusinessTech, 2020a,c; Kahla, 2021; Louw, 2021).

14. Civil society spaces that were created during the pandemic ranged from neighbourhood-level ‘Community Action Networks’ to the internationally linked People’s Vaccine Coalition (C19 People’s Coalition, 2021; van Ryneveld *et al*., 2022).

15. Organizations with a history of research and/or advocacy for evidence-based alcohol harm reduction interventions included the SAMRC, the DG Murray Trust and the SAAPA.

16. The Ministers of Transport and Police supported the alcohol regulations but presented the rationale in relation to reduced road traffic accidents and crime rather than health system benefits, while the Minister of Finance opposed the regulations due to revenue losses (Masuabi, 2020; Zulu, 2020; Ngqangashe *et al*., 2021). Several national and provincial government actors expressed support for more restrictive long-term measures, detracting from the crisis-specific purpose of the intervention (Masuabi, 2020; Gerber, 2020a).

17. The Minister of COGTA actively supported the restriction of alcohol availability during the crisis in government deliberations (Cele *et al*., 2020; Felix, 2020; Hunter, 2020). Several participants attributed her awareness of alcohol-related harms to her background as a medical doctor and former Minister of Health from 1994 to 1999 (Interviews 4, 5 and 6, 2022).

18. Public health evidence also featured prominently in the court challenge against the tobacco ban raised by British American Tobacco South Africa (BATSA). Several months after the tobacco ban was lifted, the Western Cape High Court ruled that the ban was unconstitutional due to a lack of scientific evidence. However, the court acknowledged the challenges of responding with urgency to a novel virus, and exempted the state from paying BATSA’s costs (Buthelezi, 2020).

19. Initial COVID-19 regulations were imposed for a period of 8 days. This included the closure of international borders, the closure of schools and restrictions on social gatherings.

20. Partial restrictions on alcohol sales included time-based restrictions (limitation on the days and hours during which alcohol sales were permitted), and restricted availability across different types of alcohol outlets (with specific limitations on alcohol outlets offering on-site consumption).

21. A wave of COVID-19 represents a period of increased transmission. In SA, a COVID-19 wave is defined as ‘the period from when COVID-19 weekly incidence is equal to or greater than 30 cases per 100 000 persons until the weekly incidence equal or below 30 cases per 100 000 persons’. The onset and duration of waves varied across provinces (Gage *et al*., 2024).

22. Alcohol retailers were still required to comply with regulations that did not specifically target alcohol availability, including a nationwide 11 pm curfew.

## Reflexivity statement

This study was conducted by a team of three researchers: two females and one male, spanning multiple levels of seniority, and all currently based in South Africa. Two of the authors have experience in research activities related to alcohol harms and policy in South Africa, with a health systems and policy focus. Three authors have experience conducting qualitative research.

The three contributing authors in this study all work or are engaged in studies in the field of Public Health and bring this lens to this research. We have, however, sought to limit any bias and maintain objectivity in the research by applying the principles of rigour in qualitative research.
