# Supplementary material for: The influence of crisis on policy formulation: the case of alcohol regulation in South Africa during COVID-19 (2020–21)
Source: Health Policy Plan. 2024 Jun 28;39(7):753–70. doi: 10.1093/heapol/czae055 (PMC11308613; doi:10.1093/heapol/czae055)
Supplement: czae055_Supp [file czae055_supp.zip › Supplementary data.docx]

## Supplementary data: List of documents

Data included 127 documents, including media and communications (n=74) (Table 1), official documents (n=11) (Table 2), working documents (n=8) (Table 3), legal documents (n=4) (Table 4), and scholarly works (n=30) (Table 5) ^[[1]](#footnote-1)^. The majority of these documents are dated between January 2020 and December 2021. Five documents that pre-dated January 2020 were included, as they were relevant to understanding the pre-pandemic context for alcohol regulation in SA. Six documents that post-dated December 2021 were included, as they were relevant to the study time period but were only published thereafter.

**Table 1.** Media and communications

| **Date** | **Title** | **Author** | **Publication** |
| --- | --- | --- | --- |
| 2020/04^[[2]](#footnote-2)^ | MPs debate Parliament’s role during coronavirus pandemic | Mava Lukani | In session: Official newspaper of the Parliament of the Republic of South Africa |
| 2020/04/05 | Bheki Cele: ‘I wish alcohol ban could be extended beyond lockdown’ | Queenin Masuabi | City Press |
| 2020/04/14 | Gauteng Liquor Board to take Ramaphosa to court over alcohol ban | Staff writer SABC | SABC News |
| 2020/04/18 | SA's battle of the booze explained | Jan Gerber | News24 |
| 2020/04/28 | Could South Africa’s lockdown ‘experiment’ help chart a path to a more sober and less violent country? | Koot Kotze | Bhekisisa M&G |
| 2020/04/30 | Mboweni ‘didn’t like ban on alcohol, cigarette sales’, but ‘lost the debate’ | Makhosandile Zulu | The Citizen |
| 2020/05/07 | National Coronavirus Command Council: Who guards the Guardians? | Ferial Haffajee | Daily Maverick |
| 2020/05/11 | 21 lockdown admission of guilt fines (a list of penalties for each offence) | Kabous le Roux | CapeTalk |
| 2020/05/14 | Buying alcohol in South Africa could be very different at level 3 – here are new proposals by the liquor association | Staff writer | BusinessTech |
| 2020/05/16 | South African drink makers meet with government to discuss how to resume trading | Bloomberg | BusinessTech |
| 2020/05/22 | Lockdown: Dlamini-Zuma pushes for tobacco, alcohol ban to continue until Level 1 | Qaanita Hunter | News24 |
| 2020/05/24 | Draft level 3 regulations allow alcohol sales, but renew tobacco ban | S'thembile Cele, Andisiwe Makinana, Thabo Mokone, Aphiwe de Klerk | Sunday Times |
| 2020/05/27 | Minimising health risks under Covid – is easing the ban on alcohol the right move? | Maurice Smithers | Daily Maverick |
| 2020/06^[[3]](#footnote-3)^ | Covid-19 has laid bare the inequalities of South African society | Abel Mputing | In session: Official newspaper of the Parliament of the Republic of South Africa |
| 2020/06/12 | Gauteng to revisit alcohol ban | Staff writer | BusinessTech |
| 2020/06/13 | OPINION \| Understanding the Disaster Management Act and its implementation | Dewald van Niekerk; Elmien du Plessis | News24 |
| 2020/06/15 | Lockdown: Trust in Ramaphosa down by almost 10%, while 63% say no to booze sales – survey | Unnamed reporter | Daily Maverick |
| 2020/06/16 | Malema calls for return of ban on booze throughout lockdown | Zingisa Mvumvu | TimesLive |
| 2020/06/22 | Interest groups propose 5 urgent measures to curb alcohol abuse in SA | Qama Qukula | CapeTalk |
| 2020/06/27 | As restaurants re-open, lobbying for on-site alcohol consumption will continue | Paula Luckhoff | CapeTalk |
| 2020/06/29 | Restaurants slap DTI with legal ultimatum on ban on wining while dining | Barbara Friedman | CapeTalk |
| 2020/06/30 | Level 3: Trade minister ‘pressured to lift alcohol ban’ in restaurants | Tom Head | Unassigned |
| 2020/07/03 | UI COVID-19 Webinar 15: South Africa's response to Covid-19: trajectory of the pandemic | Charles Parry | YouTube |
| 2020/07/03 | Stop shifting blame to drinkers: lobby groups to alcohol industry | Sipokazi Fokazi | TimesLive |
| 2020/07/13 | Mkhize defends alcohol ban: 'Every bed, every healthcare worker, every once of oxygen is needed' | Alex Mitchley | News24 |
| 2020/07/13 | Coronavirus: South Africans divided over second alcohol ban | Pumza Fihlani | BBC News |
| 2020/07/14 | Alcohol ban is putting a band aid on a festering wound, says ministerial advisor | Qama Qukula | CapeTalk |
| 2020/07/14 | Trauma cases leapt 62% when first booze ban ended, says Western Cape | Aron Hyman | TimesLive |
| 2020/07/14 | The data behind the alcohol ban | Department of Health | Twitter |
| 2020/07/17 | 67 bears with a purpose | George Herald | George Herald |
| 2020/07/24 | At least four arrested during #ServeUsPlease protest | Ashraf Hendricks; Zoe Postman | GroundUp |
| 2020/08/03 | Booze ban: SAMRC professor hits back at alcohol industry critics of lockdown law | Alex Mitchley | News24 |
| 2020/08/07 | Alcohol sales ban is causing corporate carnage: Agri SA | Puleng Modupe | SABC News |
| 2020/08/12 | Ramaphosa told to lift cigarette, alcohol ban and move to Level 2 lockdown - sources | Qaanita Hunter | News24 |
| 2020/08/17 | SA’s lockdown liquor ban: All the spats and stats | Joan van Dyk | Bhekisisa |
| 2020/08/17 | 'I do not run government. I am part of a collective' - Dlamini-Zuma on alcohol, tobacco decisions | Jason Felix | News24 |
| 2020/08/18 | OPINION \| Unbanning booze sales: Did we do it the right way? | Charles Parry | Bhekisisa M&G |
| 2020/08/19 | Collins Khosa murder: Military ombud finds that soldiers acted improperly | Andisiwe Makinana | TimesLive |
| 2020/08/21 | Zweli Mkhize \| The burden of alcohol on the healthcare system | Zweli Mkhize | News24 |
| 2020/08/26 | Liquor industry supports government’s fight against alcohol abuse | Staff writer SABC | SABC News |
| 2020/12/11 | Tobacco sales ban was unconstitutional and unnecessary, court finds | Londiwe Buthelezi | News24 |
| 2020/12/27 | North West Health MEC calls for ban on alcohol sales | Patrick Dintwa | SABC News |
| 2021/01/01 | Historic first: Chris Hani Baragwanath Hospital trauma unit empty on New Year's Eve | Alex Mitchley | News24 |
| 2021/01/11 | AB InBev’s legal battle over alcohol ban could be a presage of more to come | Sasha Planting | Daily Maverick |
| 2021/01/11 | SAB stands behind its booze campaign | Lindile Sifile | Sowetan Live |
| 2021/01/12 | Towards a People's Vaccine Campaign A call to action | C19 People's Coalition | Unassigned |
| 2021/01/15 | Ramaphosa 'obviously concerned' about SAB divestment, impact on growth | Carin Smith | News24 |
| 2021/01/27 | South Africa's wine industry heads to court to ﬁght alcohol ban | Tanisha Heiberg | Reuters |
| 2021/01/31 | Government moots lifting booze ban | Jan Gerber | News24 |
| 2021/02/01 | Booze ban likely to be lifted — with limits Booze ban likely to be lifted — with limits | Lizeka Tandwa | Mail & Guardian |
| 2021/02/10 | Exposed: EU Invites Big Alcohol, Offers Funding to Interfere in South Africa's Alcohol Policy Development | Movendi International | Movendi International website |
| 2021/02/17 | Cape Town moves ahead with new alcohol laws | Staff writer | BusinessTech |
| 2021/04/07 | Lobby groups try to claim booze ban not linked to drop in trauma cases | Sasha Planting | Daily Maverick |
| 2021/04/08 | New research highlights major flaws in South Africa’s lockdown alcohol bans | Staff writer | BusinessTech |
| 2021/04/16 | Why South Africa banned booze - And what happened next | Eyder Peralta | NPR |
| 2021/04/28 | 'Disturbing and detrimental'- alcohol industry fumes over no health recommendation for third ban | Penelope Mashego | News24 |
| 2021/05/09 | Thank you for drinking: Alcohol industry uses tobacco playbook to counter lockdown bans | Mark Tomlinson | Daily Maverick |
| 2021/05/24 | Alcohol restrictions and any ban must be rejected | Dean Macpherson | DA website |
| 2021/05/24 | Third Covid wave: Booze ban not off the table, says Health Dept | Rorisang Kgosana | The Citizen |
| 2021/06/18 | Here’s how many liquor stores these retailers have in SA, and how many they opened despite Covid-19 | Phumi Ramalepe | Business Insider |
| 2021/06/27 | Alcohol ban: Here’s what government could do instead | Cheryl Kahla | The Citizen |
| 2021/06/27 | Ramaphosa announces fourth alcohol ban as SA heads into Level 4 lockdown | Ahmed Areff | News24 |
| 2021/06/28 | Liquor Traders Council warns that members are considering defying the ban on alcohol sales | Risha Maduray | SABC News |
| 2021/07/03 | Alcohol bans reduced deaths, study finds | Unnamed reporter | GroundUp |
| 2021/07/06 | Booze industry pleads for relief as possible sales ban extension looms | Penelope Mashego | News24 |
| 2021/07/12 | Booze ban: The abuse – not the use – of alcohol needs a more realistic and sober solution | Ingrid Louw | Daily Maverick |
| 2021/07/22 | Court dismisses SAB's bid against booze sales ban | Unnamed reporter | News24 |
| 2021/07/22 | R500m worth of looted alcohol now on the streets, being sold in illicit market | Staff writer | TimesLive |
| 2021/08/23 | Court case to decide on alcohol sales in South Africa | BusinessTech staff writer | BusinessTech |
| 2021/08/31 | BP is the first petrol station in South Africa to be given a liquor licence – and already faces calls for it to be blocked | Staff writer | BusinessTech |
| 2021/09/21 | 'Liquor Products Bill signed into law, but it doesn't tackle SA's booze problem' | Qama Qukula | CapeTalk |
| 2021/10/20 | Pick n Pay's Ackerman concerned that elections will bring new booze sales ban | Penelope Mashego | News24 |
| 2021/12/07 | Alcohol bans were necessary to manage Covid-19 pandemic, court rules in Vinpro case | Carin Smith | News24 |
| 2022/01/12 | SAB 'disappointed' after it's denied leave to appeal alcohol ban case | Carin Smith | News24 |

**Table 2.** Official documents

| **Date** | **Title** | **Author** | **Publication** |
| --- | --- | --- | --- |
| 2020/03/23 | Statement by President Cyril Ramaphosa on escalation of measures to combat COVID-19 epidemic | President Cyril Ramaphosa | SA government official website |
| 2020/04/17 | Ban on sale of alcohol to remain in force | Presidency of the Republic of SA | COVID-19 Online resource & news portal |
| 2020/04/23 | President Cyril Ramaphosa: South Africa's response to Coronavirus COVID-19 pandemic | President Cyril Ramaphosa | COVID-19 Online resource & news portal |
| 2020/05/20 | Health Minister’s statement on Prof Glenda Gray’s public attack of government based on inaccurate information | Minister of Health, Dr Zweli Mkhize | COVID-19 Online resource & news portal |
| 2020/05/28 | Minister Nkosazana Dlamini Zuma: Coronavirus Covid-19 Level 3 Lockdown Regulations | Minister of COGTA, Dr Nkosazana Dlamini Zuma | COVID-19 Online resource & news portal |
| 2020/06/17 | Address by President Cyril Ramaphosa on South Africa’s response to the coronavirus pandemic | President Cyril Ramaphosa | Official website of the presidency of SA |
| 2020/07/12 | Statement by President Cyril Ramaphosa on progress in the national efforts to contain the COVID-19 pandemic | President Cyril Ramaphosa | Official website of the presidency of SA |
| 2020/07/13 | Social Cluster media briefing 13 July | Department of Health | COVID-19 Online resource & news portal |
| 2021/01/11 | Statement by President Cyril Ramaphosa on progress in the national effort to contain the Covid-19 pandemic, 11 January 2021 | President Cyril Ramaphosa | Official website of the presidency of SA |
| 2021/01/26 | MEC Ivan Meyer calls for lifting of alcohol ban | Ivan Meyer | SA government official website |
| 2021/07/11 | President Cyril Ramaphosa: Progress in national effort to contain the Coronavirus COVID-19 pandemic | President Cyril Ramaphosa | SA government official website |
| 2021/11/28 | Statement by President Cyril Ramaphosa on progress in the national effort to contain the COVID-19 pandemic, 28 November 2021 | President Cyril Ramaphosa | COVID-19 Online resource & news portal |

**Table 3.** Working documents

| **Date** | **Title** | **Author** | **Publication** |
| --- | --- | --- | --- |
| 2020/07/09 | Presentation to the National Coronavirus Command Council (PowerPoint slides) | Professor Salim S. Abdool Karim | COVID-19 Online resource & news portal |
| 2020/07/22 | Advisory on the NCCC questions on actions to curb the surge (Internal memo from Ministerial Advisory Committee (MAC) on COVID-19 to Minister Z Mkhize) | Professor Salim S. Abdool Karim | COVID-19 Online resource & news portal |
| 2020/07/22 | Advisory on the NCCC questions on actions to curb the surge: Attachment 4 - Considerations on alcohol restrictions | Technical working group on alcohol | COVID-19 Online resource & news portal |
| 2020/12/23 | Mitigating the spread of SARS-COV-2, including the new Coronavirus variant, and preserving the healthcare system’s capacity (Internal memo from Ministerial Advisory Committee (MAC) on COVID-19 to Minister Z Mkhize) | Professor Salim S. Abdool Karim | COVID-19 Online resource & news portal |
| 2021/03/15 | A qualitative analysis of comments received from the general public in response to gazetted amendments to the Disaster Management Act Regulations: Alert Level 3 (Gazette 43521 of 12 July 2020). (Commissioned by the Minister of COGTA, Dr Nkosazana Dlamini-Zuma, August) | Reddy, P., Soudien, C., Shean, Y., Mjimba, V., Oppelt, T., Fluks, L., Magampa, M., Houston, G., Ntlangula, M., Coert, S. & Sobane, K. | Human Sciences Research Council |
| 2021/09/23 | Mitigating the impact of COVID-19 during the municipal elections – update (Internal memo from Ministerial Advisory Committee (MAC) on COVID-19 to Minister MJ Phaahla) | Professor Koleka Mlisana and Prof Marian Jacobs | COVID-19 Online resource & news portal |
| 2021/11/01 | Appeal Hearing Decision: SAAPA vs Sunday Times | Professor K Govender (Appeals Panel) | Official website of the Press Council |

**Table 4.** Legal documents

| **Date** | **Title** | **Author** | **Publication** |
| --- | --- | --- | --- |
| 2004/04/26 | No. 59 of 2003: Liquor Act, 2003 | N/A | Government Gazette No. 26294 |
| 2020/03/15 | Disaster Management Act, 2002. Declaration of a National State of Disaster | Department of Co-operative Governance and Traditional Affairs | Government Gazette No. 43096 |
| 2020/03/18 | Regulations issued in terms of Section 27(2) of the Disaster Management Act, 2002. | Department of Co-operative Governance and Traditional Affairs | Government Gazette No. 43107 |
| 2020/03/25 | Directions issued by the Minister of Co-operative Governance and Traditional Affairs with respect to the response to COVID-19 in the Co-operative Governance and Traditional Affairs sectors | Department of Co-operative Governance and Traditional Affairs | Government Gazette No. 43147 |

**Table 5.** Scholarly work

| **Date** | **Title** | **Type of work** | **Author** | **Publication** |
| --- | --- | --- | --- | --- |
| 2005 | Crisis Policy Making and Management in Southeast Asia | Book chapter | Scott Fritzen | Encyclopaedia of Public Administration and Public Policy |
| 2010/11 | Inequitable access to substance abuse treatment services in Cape Town, South Africa | Empirical research | Bronwyn J Myers, Johann Louw, Sonja C Pasche | Substance Abuse Treatment, Prevention and Policy |
| 2014 | A critical analysis of the South African Disaster Management Act and Policy Framework | Critical analysis | Dewald van Niekerk | Disasters |
| 2018 | Unpacking policy formulation and industry influence: the case of the draft control of marketing of alcoholic beverages bill in South Africa | Empirical research | Adam Bertscher, Leslie London, and Marsha Orgill | Health Policy and Planning |
| 2020/05 | Alcohol use in times of the COVID 19: Implications for monitoring and policy | Commentary | Jürgen Rehm, Carolin Kilian, Carina Ferreira-Borges,  David Jernigan, Maristela Monteiro, Charles D. H. Parry, Zila M. Sanchez & Jakob Manthey | Drug and Alcohol Review |
| 2020/05/14 | How South Africa’s Ministerial Advisory Committee on COVID-19 can be optimised | Editorial | Jerome Amir Singh | South African Medical Journal |
| 2020/07/15 | Prohibiting alcohol sales during the coronavirus disease 2019 pandemic has positive effects on health services in South Africa | Empirical research | Hermann Reuter, Louis S. Jenkins, Marischka De Jong, Steve Reid, Michael Vonk | African Journal of Primary Health Care & Family Medicine |
| 2020/09 | What Is COVID-19 Teaching Us About Community Health Systems? A Reflection From a Rapid Community-Led Mutual Aid Response in Cape Town, South Africa | Perspective | Manya van Ryneveld, Eleanor Whyle, Leanne Brady | International Journal of Environmental Research and Public Health |
| 2020/11 | Letter to Editor South Africa’s COVID-19 Alcohol Sales Ban: The Potential for Better Policy-Making | Letter to the editor | Richard Matzopoulos,  Helen Walls, Sarah Cook, Leslie London | International Journal of Health Policy and Management |
| 2020/11 | Trauma patterns during the COVID-19 lockdown in South Africa expose vulnerability of women | Empirical research | A Zsilavecz, H Wain, L J Bruce, M T D Smith, W Bekker, G L Laing, E Lutge, D L Clarke | South African Medical Journal |
| 2021 | Controversy in the Time of COVID: A Qualitative Content Analysis of Three Case Studies from South African Online News Sites | Empirical research | Ruth Teer-Tomaselli | The Political Economy of Communication |
| 2021/01 | The burden of alcohol on health care during COVID-19 | Commentary | Tim Stockwell, Sven Andreasson, Cheryl Cherpitel, Tanya Chikritzhs, Frida Dangardt, Harold Holder, Timothy Naimi & Adam Sherk | Drug and Alcohol Review |
| 2021/01 | A timely piece that resonates with the South African experience: Commentary on Stockwell et al. | Commentary | Charles D H Parry | Drug and Alcohol Review |
| 2021/02 | Alcohol industry arguments for putting proﬁt before health in the midst of a pandemic: The Western Australian experience | Commentary | Danica Keric & Julia Stafford | Drug and Alcohol Review |
| 2021/02 | The effect of lockdown on intentional and nonintentional injury during the COVID-19 pandemic in Cape Town, South Africa: A preliminary report | Empirical research | P H Navsaria, A J Nicol,  C D H Parry, R Matzopoulos, S Maqungo, R Gaudin | South African Medical Journal |
| 2021/03 | Alcohol consumption, harms and policy developments in sub-Saharan Africa: The case for stronger national and regional responses | Review | Neo K. Morojele, Emeka W. Dumbili , Isidore S. Obot & Charles D. H. Parry | Drug and Alcohol Review |
| 2021/04/20 | Social policy as an integral component of pandemic response: Learning from COVID-19 in Brazil, Germany, India and the United States | Empirical research | Scott L. Greer, Holly Jarman, Michelle Falkenbach, Elize Massard da Fonseca, Minakshi Raj & Elizabeth J. King | Global Public Health: An International Journal for Research Policy and Practice |
| 2021/05/17 | Trauma trends during COVID-19 alcohol prohibition at a South African regional hospital | Empirical research | Kathryn M. Chu, Jenna-Lee Marco, Eyitayo Omolara Owolabi, Riaan Duvenage, Mukhethwa Londani, Carl Lombard, Charles D. H. Parry | Drug and Alcohol Review |
| 2021/07/29 | What lessons does the COVID-19 pandemic hold for global alcohol policy? | Commentary | June Yue Yan Leung, Shiu Lun Au Yeung, Tai Hing Lam, Sally Casswell | BMJ Global Health |
| 2021/09 | Unnatural deaths, alcohol bans and curfews: Evidence from a quasi-natural experiment during COVID-19 | Empirical research | T A Moultrie, R E Dorrington, R Laubscher, P Groenewald, C D H Parry, R Matzopoulos, D Bradshaw | South African Medical Journal |
| 2021/10 | Regulating Alcohol: Strategies Used by Actors to Inﬂuence COVID-19 Related Alcohol Bans in South Africa | Empirical research | Yandisa Ngqangashe, Maddie Heenan and Melanie Pescud | International Journal of Environmental Research and Public Health |
| 2021/12/08 | Intersectoral collaboration before and during the COVID-19 pandemic in the Western Cape: implications for future whole-of-society approaches to health and wellbeing | Empirical research | Helen Schneider, Ida Okeyo, Alheit du Toit, Beth Engelbrecht Leslie London, Elizabeth Pegram Gavin Reagon, Keith Cloete | South African Health Review |
| 2021/12/21 | Which Rights? Whose Rights? Public Health and Human Rights through the Lens of South Africa’s COVID-19 Jurisprudence | Peer-reviewed journal publication | Safura Abdool Karim & Petronell Kruger | Constitutional Court Review |
| 2021/12 | Sobering up in South Africa: The Sin Tax Consequences of a Pandemic | Review | Teresa Pidduck & Sumarie Swanepoel | New Zealand Journal of Taxation Law and Policy |
| 2022 | South Africa: Surfing towards centralisation on the Covid-19 wave | Book chapter | Nico Steytler, Jaap de Visser and Tinashe Chigwata | Comparative Federalism and COVID-19: Combating the pandemic |
| 2022/02/17 | When secrecy and expert advice collide in a pandemic: Access to information and the National Department of Health’s tardy publication of Ministerial Advisory Committee advisories | Editorial | Marlise Richter, Yanga Nokhepheyi, Fatima Hassan | South African Medical Journal |
| 2022/03 | The cost of harmful alcohol use in South Africa: A commentary | Correspondence | M Murray & G D I Barr | South African Medical Journal |
| 2022/03/12 | Does international trade and investment liberalization facilitate corporate power in nutrition and alcohol policymaking? Applying an integrated political economy and power analysis approach to a case study of South Africa | Empirical research | Penelope Milsom, Richard Smith, Simon Moeketsi Modisenyane and Helen Walls | Globalization and Health |
| 2022/04 | Changes in retrospectively recalled alcohol use pre, during and post alcohol sales prohibition during COVID pandemic in Botswana | Empirical research | J. Maphisa Maphisa & Kefentse Mosarwane | International Journal of Drug Policy |
| 2022/07/01 | The cost of harmful alcohol use in South Africa: A reply to Murray and Barr (2022) | Right of reply | Richard Matzopoulos, Sarah Truen, Brett Bowman, Joanne Corrigall | South African Medical Journal |

1. Documents categories are derived from the ‘READ’ approach to document analysis in health policy research by Dalglish et al (2020). [↑](#footnote-ref-1)
2. Exact date not recorded on document [↑](#footnote-ref-2)
3. Exact date not recorded on document [↑](#footnote-ref-3)
